# Medical practitioner compassion: Development and validation of a compassion competency questionnaire

**DOI:** 10.4102/ajopa.v7i0.170

**Published:** 2025-06-12

**Authors:** Michelle Jäckel-Visser, Carl C. Theron, Robert J. Mash

**Affiliations:** 1 Department of Industrial Psychology, Faculty of Economic and Management Sciences, Stellenbosch University, Stellenbosch, South Africa; 2 Department of Family and Emergency Medicine, Faculty of Medicine and Health Sciences, Stellenbosch University, Stellenbosch, South Africa

**Keywords:** compassion, competency, measure, medical practitioners, healthcare, structural equation modelling, measurement model, MPCCQ

## Abstract

There is a need for a psychometrically robust questionnaire measuring medical practitioner compassion in healthcare. Without such a measure, competence on this construct cannot be assessed, nor can the effectiveness of interventions designed to enhance compassion be determined. The aim of this study was to develop and validate a self-assessment measure of medical practitioner compassion competence (the Medical Practitioner Compassion Competency Questionnaire [MPCCQ]). The MPCCQ was administered to a sample of 234 medical practitioners in South Africa. They represented three healthcare system levels, namely, the primary level (healthcare centres and clinics), the secondary level (district and regional hospitals) and the tertiary level (central, specialised and sub-specialist hospitals). The quantitative data were analysed with statistical packages, namely, Statistical Package for the Social Sciences (SPSS) version 25 and LISREL 8.8, and structural equation modelling was used to fit the MPCCQ measurement model and structural model. Dimensionality and item analyses returned generally positive results. Fit statistics and criteria used to judge the fit of the models included Chi-square (χ^2^), goodness of fit index (GFI), adjusted goodness of fit index (AGFI), root mean square error of approximation (RMSEA), root mean square residual (RMR) and the standardised root mean square residual (SRMR). The results provided an excellent model fit for both the measurement and comprehensive LISREL models. The MPCCQ, measurement and structural model parameter estimates supported the position that the design intention underpinning the MPCCQ succeeded.

**Contribution:**

The statistical evidence generated thus far failed to refute the position that the MPCCQ shows construct validity, thus paving the way for the cautious utilisation of the instrument in healthcare and medical education institutions.

## Introduction

The measurement of medical practitioner compassion is a topic that still seems to be evolving in the medical field (Roberts et al., 2019; Sinclair et al., 2017). As Strauss et al. (2016, p. 15) stated: ‘A new measure of compassion with robust psychometric properties is needed’. The creation of a medical practitioner metric is needed that comprehensively measures a practitioner’s compassion, drawing input from practitioners themselves, patients, peers and staff. Such a measure would be expected to assist in improving patient satisfaction and medical practitioner well-being (Zeller et al., 2024). There is no evidence of there being psychometrically sound instruments that measure compassion as a competency as displayed by medical practitioners.

However, there is evidence from researchers who have attempted to measure compassion, using empathy scales, where patients have evaluated medical practitioner compassion from an empathy point of view (Baguley et al., 2022; Malenfant et al., 2022; Snyderman & Gyatso, 2019). To address this gap, firstly, it is necessary to conceptualise compassion from a medical practitioner’s point of view, thus utilising medical practitioners as co-researchers because they are subject matter experts on the job content themselves. Secondly, a measurement tool based on this conceptualisation should be developed as a self-reflective instrument, allowing medical practitioners to evaluate themselves first, before peers, patients and allied workers evaluate them. The World Health Organization (WHO) states that clinicians should be competent and provide the highest quality of care to individuals and communities (WHO Guidelines, 2013). Therefore, instruments are needed to measure medical practitioner competence, as part of performance management, to enhance work outcomes such as high-quality patient care.

Organisations are constructed entities that exist to serve society. These entities include healthcare organisations in the public and private sectors that render important healthcare services to the public. Employee work performance determines how successful these entities are. This includes the performance of medical practitioners who play a pivotal role in the rendering of healthcare services. There should thus be focused and purposeful efforts to monitor and enhance the performance of medical practitioners. This is only possible if there is a clear understanding of what constitutes medical practitioner performance and if it can be validly measured. For this article, performance is conceptualised as a set of structurally interrelated latent behavioural competencies that map onto a set of structurally interrelated latent outcome variables. Medical practitioner performance is more than the narrow technical tasks described by the *Health Professions Act of 1974* (Republic of South Africa, 1974) and comprises more than the outcome of successfully treating a disease. Medical practitioner compassion is an example of such a competency that structurally links to latent outcome variables such as patient satisfaction (Zeller et al., 2024). Medical practitioner compassion is also important for a holistic approach to healthcare but is nonetheless currently undervalued and neglected (Visser, 2020).

Historically, early medical approaches were characterised by taking an unstructured account of a patient’s complaint, as was done in the 19th century, or by only making notes of physical signs in the chest when the Laennec stethoscope was introduced during the 19th century. A consultation was characterised by taking a history of a present complaint, noting past illnesses and family history, using a systems review and then communicating a diagnosis and prescribing treatment (Stewart & Roter, 1989). This biomedical approach was typified by poor integration of mental, emotional, social and spiritual dimensions of care (Joyner et al., 2014). In the following years, the more holistic bio-psychosocial approach was introduced (McWhinney, 1972; Stewart et al., 2005), but it still did not include dimensions such as spirituality and morality. Therefore, a more holistic approach seemed like the answer, where patients could find comfort in their medical practitioner being there and providing a safe space in which issues beyond physical concerns could be addressed (Gwyther, 2011). This approach also supports schooling in humanities and behavioural sciences. Competent medical practitioners are those who identify with the patient and spend time with them to understand both clinical problems and the patient’s life story while maintaining detached calmness and self-control but still showing compassion (Gwyther, 2011).

The display of medical practitioner compassion holds benefits for the patient (Baguley et al., 2022). Baguley (2022) refers to the following benefits to justify their belief that medical practitioner compassion is essential to effective medical care:

In medicine, compassion is desired by patients, mandated by medical regulatory bodies and increasingly linked to positive outcomes for patients and families, professionals and healthcare systems. Patients and families rate compassion among the most important healthcare requirements, recalling it years later. Compassionate care predicts faster recovery, greater autonomy, lower intensive care utilization and more responsible healthcare management. Similarly, compassion-related constructs have been associated with objective benefits, including better disease control and reduced metabolic complications among patients with diabetes. Compassion is thus central to both the practice of effective medicine and essential in the preferences of those receiving professional care. (p. 1692)

It can, however, also be argued that medical practitioners, and just about everybody else in all walks of life, need to display compassion to fully realise their human potential to become who they really are. This is not only the case in South Africa but also in the rest of the world. Maslow (1971) developed the position shortly before his death that the highest level of human motivation would be positioned on the sixth level in his six-level hierarchy of needs pyramid: self-transcendence. People on this level are involved ‘in a cause outside of their skin: in something outside of themselves, some calling or vocation’ (Maslow, 1971, p. 45). Self-transcendence is motivated by ‘being values’, and although not explicitly referring to compassion as such a value, this research argues that it could be seen as an expression of ‘goodness’ (Maslow, 1971, p. 138). Schopenhauer, a Western philosopher, supports this by emphasising that compassion is the basis of morality and should be included in health education. As much as caring is an activity that can be learnt, perceiving the moral dimension of medical practice is also categorised as a competency that can be learned (Fotaki, 2015).

A compassionate clinical encounter between doctor and patient is aligned with a holistic approach to patient care. Compassion at its core is when insight or appreciation into suffering translates into context-appropriate helping behaviour because of a generalised concern for a fulfilling life (Visser, 2020).

### Measuring medical practitioner compassion

There is no evidence of existing medical practitioner compassion competency measures in the literature. While diagnosis and treatment are measured, compassionate care is not (Zeller et al., 2024). On an international level, limited research indicates that there are 41 instruments available measuring facets of compassion such as empathy or trust. Only six instruments measure compassion as a scale or subscale, such as the Instrumental Caring Inventory (Donius, 1994); the Disposition Positive Emotions Scale (Shiota et al., 2006); Fears of Compassion Scale (Gilbert et al., 2011); Neuroticism, Extraversion, Openness Personality Inventory Revised (psychiatry) (NEO PI-R) Standard (Costa & McCrae, 2008); the Santa Clara Brief Compassion Scale (SCBCS) (Hwang et al., 2008); and the Professional Quality of Life Scale (ProQOL) (Sprang et al., 2007). Some of these scales are reported to have reliable psychometric properties, such as the Compassion Satisfaction and Fatigue Test, with an alpha reliability coefficient of 0.87 for the compassion fatigue subscale (Bride et al., 2007), and the ProQOL with alpha reliability coefficients of 0.87 for the compassion satisfaction subscale (Stamm, 2005). None of these instruments measures compassion for a medical practitioner *per se* (Visser, 2020). A systematic search of compassion measures by Strauss et al. (2016) has shown that all identified measures had notable psychometric weaknesses, confirming the need for a rigorous measure that can be used in healthcare. Without proper measurements, it is not possible to determine competence on this construct or whether training interventions designed to enhance medical practitioner compassion are effective.

The manner in which medical practitioners tend to their patients is important. The current study is concerned that compassion is being neglected in healthcare, not only in the public healthcare sector in South Africa but worldwide. If this concern is justified, it calls for corrective action to restore compassion to its rightful place when rendering holistic care (bio-psychosocial model) to patients. In terms of this line of reasoning, there is a need for a valid and reliable instrument to measure medical practitioner compassion that allows for assessing behavioural performance and evaluating this competency as part of the medical practitioner performance construct.

The aim of this article is to develop the Medical Practitioner Compassion Competency Questionnaire (MPCCQ) and to empirically test whether it provides a reliable and construct-valid measure of medical practitioner compassion as constitutively defined. To successfully operationalise the compassion construct, it is important to first obtain an accurate conceptual grasp on the construct of medical practitioner compassion.

### Conceptualising compassion

To manage and accurately measure compassion, it should be clear what constitutes compassion. Compassion is a construct which is essentially an abstract idea that is assigned a specific connotative meaning for an explanatory (or descriptive) purpose. A construct like compassion does not have an absolute connotative meaning. The current study attached a specific connotative meaning to the medical practitioner compassion construct grounded in the literature-based hypothesis that when medical practitioners in the public healthcare sector in South Africa (and elsewhere in the world) display competence in the compassion competency as conceptualised in this study, it will be instrumental in positive patient outcomes (Lown, et al., 2015; Spandler & Stickley, 2011). The connotative meaning attached to a construct may be regarded as theoretically valid if it is aligned with the manner in which it is used in explanations and descriptions (Mouton & Marais, 1985). The current study acknowledges that the manner in which patients from different cultures in South Africa conceptualise compassion might be different from the manner in which the researchers conceptualised compassion. Probably even more important is the possibility that the behavioural denotations of medical practitioner compassion may differ across patients from different cultures in South Africa. This line of reasoning underlines the importance of eventual measurement invariance and equivalence research.

The English word ‘compassion’ is derived from the Latin word *patior*, literally meaning *to suffer*. It originates from the Latin root *com* + *pati, com* meaning *with, together* and *pati* meaning to *bear, suffer*. In other words, ‘co-suffering’ (Onions et al., 1966). During conceptualisation, the internal structure (connotative meaning)^1^ was explicated before developing the indirect measure.

The connotative meaning of the *compassion* competency construct was represented as a structural model that led to a unique definition of medical practitioner compassion in healthcare. This definition consists of six latent dimensions, namely: *investing the self, mindfulness, recognition of emotions, gaining and communicating empathic understanding, caring with kindness* and *compassion action orientation*. Definitions of these six compassion competency dimensions are provided in Visser (2020). Understanding the meaning of these dimensions, as well as the way in which they are related in the structural model, led to the constitutive definition of the *compassion* competency construct used in this research article, namely (Visser, 2020):

*Recognising* someone’s suffering, attentively living in the moment of the patient-practitioner encounter, investing the self in the role of alleviating the suffering, developing and conveying an insider phenomenological *understanding* of someone’s suffering, demonstrated *authentic affection and care* for the individual and *implementing tangible context appropriate action* to alleviate the suffering. (p. 331)

**FIGURE 1:**
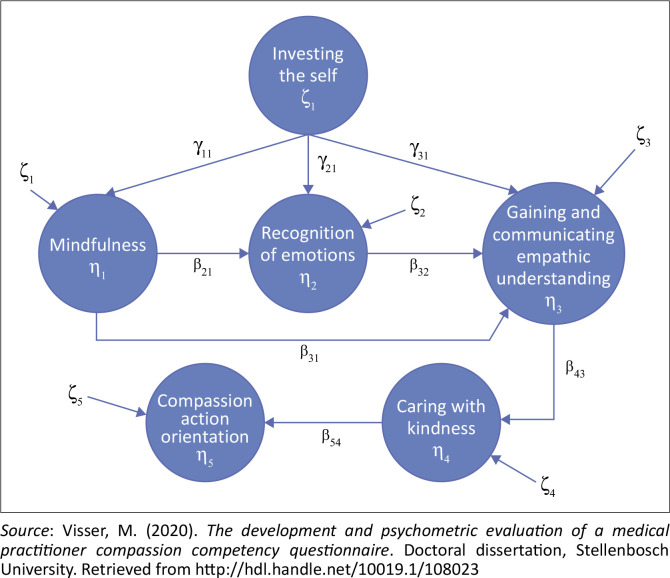
Medical practitioner compassion structural model reflecting the internal structure of the multidimensional compassion construct.

The medical practitioner compassion structural model used as a definition for this research is illustrated in Figure 1.

### Psychological measurement

The level of performance medical practitioners achieve on this competency is not an outcome of a random event but is determined by a complex nomological network of latent dimensions. To allow the monitoring of a medical practitioner’s level of competence (i.e., the extent to which a standard has been surpassed), an instrument is required that provides a reliable, construct-valid and unbiased measure of this construct. This allows one to derive construct-valid and unbiased inferences about a medical practitioner’s standing on the construct (Kurz & Bartram, 2002).

The medical practitioner compassion competency construct, as constitutively defined, was operationalised by utilising medical practitioners in the public health sector as co-researchers to generate behavioural denotation for each of the six competency dimensions. The behavioural denotations were used as cues from which to write items for the questionnaire. A draft MPCCQ was developed to measure the medical practitioner compassion competency construct based on the subject matter expert input and the researchers’ cognition. The MPCCQ was standardised to ensure that variation in test scores can be interpreted only in terms of the construct to be measured and not in terms of non-relevant contextual variables, by attempting to hold these latter variables constant across respondents and testing occasions (Anastasi & Urbina, 1997; Murphy & Davidshofer, 2005).

## Methods

This study, classified as evaluation research,^2^ aimed at the validation of the construct-referenced inferences derived from a measuring instrument. An evaluation study is set in motion by the research-initiating set of questions that ask: can the intervention be expected to achieve its intended objectives, does it achieve its objectives, and does it do so in the manner intended? (Theron, 2017; Visser, 2020). Thus, the research-initiating question for this research was: *Does the developed MPCCQ provide a reliable measure of compassion, and may inferences of the compassion construct as constitutively defined be permissibly derived from the MPCCQ scores?*

### Research design

An *ex-post facto* correlational design was used because it allows the empirical investigation of hypotheses that are frequently encountered in the social and behavioural sciences which cannot easily be investigated via experimental designs (Kerlinger & Lee, 2000). It does not allow the random assignment of observations nor the manipulation of latent variables. The correlational design allows one to observe the independent and dependent variables across individuals to determine the extent to which they co-vary (Theron, 2016; Visser, 2020).

### Study population and sample

Data were collected from medical practitioners working in the public health sector in South Africa and registered with the Health Professions Council of South Africa (HPCSA). A non-probability sample was selected using the following inclusion criteria: registration with the HPCSA as medical practitioners; practitioners had to be in clinical practice following internship and community service, and they had to practise in one of the five core disciplines: family medicine, internal medicine, paediatrics, obstetrics and gynaecology, and surgery. Lastly, participants had to represent all three healthcare levels: tertiary, secondary and primary level.

A sample of 234 medical practitioners was drawn from the public healthcare sector of South Africa. Only 178 participants specified their age; the youngest participant was 24 years old, while the eldest was 70 years old. The average age of participants was 40.85 years. The majority of the sample were female participants (53.9%), whereas male participants represented 45.7% of the sample. Concerning home language, the majority of respondents were English-speaking (46.2%), followed by Afrikaans-speaking (38.5%). Other languages included Xhosa (3.8%), Northern Sotho (1.3%), Tswana (1.3%), Tsonga (0.9%), Venda (0.9%), Sotho (0.4%), Swazi (0.4%) and Zulu (0.4%). Most participants (50.9%) came from secondary level district and regional hospitals, while 25.6% came from the tertiary level, and 21.8% from primary level care facilities. Four participants chose not to indicate the healthcare system level in which they work. The majority of participants represented the family medicine discipline (39.7%), followed by surgery (16.2%), internal medicine (13.7%), obstetrics and gynaecology (13.2%), and paediatrics (10.3%), the least represented discipline.

### Data collection instrument

The data collection instrument was the MPCCQ, developed to provide formative feedback on how medical practitioners display the performance construct of compassion during medical encounters. Test items for the instrument were developed from critical incident technique interviews (Visser, 2020), which were incorporated into the MPCCQ. The critical incidents were captured as short statements (items) on which the participants needed to rate themselves on a five-point rating scale. Response options were: significant development area (1), development area (2), on par/satisfactory (3), strength (4), and well-developed strength (5). An additional response option was included for the inability to respond. Three of the response options were anchored with behavioural descriptions of what constituted *significant development area, on par/satisfactory* and *well-developed strength*. A score was then calculated for each of the six subscales, which together made up the compassion profile (Visser, 2020). Scoring was carried out manually.

The draft questionnaire was pilot-tested with three medical practitioners. The pretest was necessary to determine if the questionnaire items were clearly formulated, and to identify problem areas so that they could be addressed before the empirical quantitative data-gathering process was initiated (Bradburn et al., 2004). Unclear terminology was clarified and a few editorial changes were made. The questionnaire comprised six subscales, each representing a specific latent dimension of compassion as a behavioural construct. Five of the six subscales comprised six items, whereas the compassion action orientation competency subscale comprised seven items (Visser, 2020).

**FIGURE 2:**
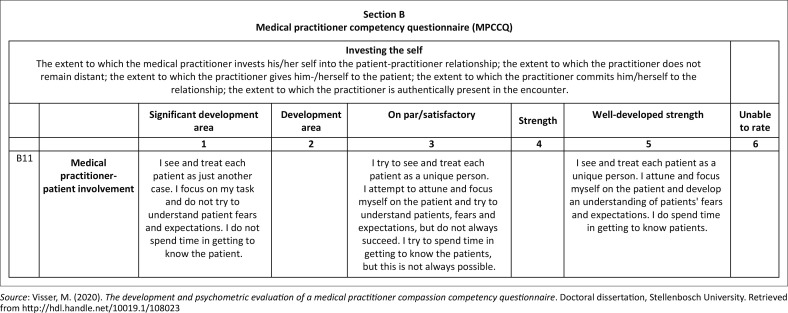
Example of medical practitioner compassion competency questionnaire items.

The final questionnaire consisted of two sections – Section A: Biographical information, and Section B: MPCCQ. There were 37 items measuring six dimensions. Completion took about 20 min – 30 min. An example of one of the items is presented in Figure 2.

### Data analysis

Classical measurement theory item statistics were calculated for each subscale via the Statistical Package for the Social Sciences (SPSS) version 25 reliability procedure, namely: item means, item standard deviations, inter-item correlations, corrected item-total correlations, squared multiple correlations, subscale mean if item deleted, subscale variance if item deleted, and subscale internal consistency reliability if item deleted (Visser, 2020). The item analysis attempted to control non-relevant human characteristics by trying to deprive them of the opportunity to influence test behaviour and to identify insensitive items that did not discriminate between the constructs of interest and could be removed (Theron, 1999). Dimensionality analysis was performed via restricted exploratory factor analysis (Tabachnick & Fidell, 2007). Reliability analysis calculated Cronbach’s alpha and McDonald’s omega for each subscale. Structural equation modelling (SEM) was used to evaluate the extent to which the design intention underpinning the MPCCQ succeeded. This was performed by fitting the measurement model that mapped the subscale items onto the six latent compassion dimensions via LISREL 8.8. Structural equation modelling was used to fit the structural model shown in Figure 1 that shows the internal structure that was attributed to the medical practitioner compassion competency construct. The following model fit indices and criteria were used to judge the fit of both these models: Chi-square (χ^2^), goodness of fit index (GFI), adjusted goodness of fit index (AGFI), root mean square error of approximation (RMSEA), root mean square residual (RMR), and the standardised root mean square residual (SRMR). For good fit, the following criteria were used: not rejecting the exact fit null hypothesis (H_0_: RMSEA = 0), not rejecting the close fit null hypothesis (H_0_: RMSEA ≤ 0.05), SRMR of less than 0.05 (Browne & Cudeck, 1993); comparative fit index (CFI) of more than 0.95 (Hu & Bentler, 1999) and a smaller Akaike information criterion (AIC) (Browne & Cudeck, 1993; Hu & Bentler, 1999; Jöreskog & Sörbom, 1993).

### Ethical considerations

Ethical approval was granted by the university’s Departmental Ethics Screening Committee (DESC). A formal ethical clearance certificate was issued by the Research Ethics Committee Human Research (Humanities), reference number IPSY-2018-6416. Informed institutional permission was obtained from the university (reference number: IRPSD891, 28 June 2018) and the Western Cape Department of Health (reference: Research Projects, dated 19 July 2018). All study participants provided written consent and were informed of the voluntary nature of the research and their right to withdraw at any time with no consequences. The MPCCQ was anonymised.

## Results

### Dimensionality analysis

Restricted exploratory factor analysis (principal axis factoring), in which the extraction of a single factor was requested, corroborated the unidimensionality of three of the subscales (*investing the self, gaining and communicating empathic understanding*, and *caring with kindness*) in that the extracted factor structure was able to reproduce the observed inter-item correlation matrices with less than 30% of the residual correlations exceeding 0.05. For three of the subscales (mindfulness, recognition of emotion and compassion action orientation), the extracted single-factor structure failed to provide a valid explanation of the observed inter-item correlation matrix. The extraction of two factors was consequently requested for these three subscales for which the unidimensionality assumption was not corroborated. First-order measurement models were fitted via confirmatory factor analysis. All three of these first-order measurement models showed acceptable fit. Second-order measurement models were subsequently fitted, in which the two first-order factors were loaded on a single second-order factor (Visser, 2020).

The loadings of the first-order factors on the second-order factor were constrained to be equal. These three second-order measurement models also showed acceptable fit. Visser (2020) did not mathematically transform the second-order measurement models into Schmid-Leiman bifactor models. Botha (2025) did so by analysing Visser’s (2020) data to determine the extent to which the themes unique to the extracted first-order factors contributed to the explained subscale item variance. Botha (2025) found that the broad, general factor, representing the variance shared by the extracted first-order factors, accounted for the majority of the explained subscale item variance in the case of all three subscales. The broad, general factor accounted for 0.9186 of the explained item variance in the *mindfulness* subscale, 0.9058 of the explained item variance in the *recognition of emotion* subscale, and 0.8280 of the explained item variance in the *compassion action orientation* subscale (Botha, 2025). Essential unidimensionality can therefore be concluded for these three subscales of the MPCCQ.

### Item analysis

Classical measurement theory item analysis was performed on each of the six subscales separately. Based on a basket of item statistics, item B16 of the *investing the self* subscale and item B67 of the *compassion action orientation* subscale were flagged as problematic items and deleted, resulting in an increase in the Cronbach’s alpha (from 0.622 to 0.669 in the case of the *investing the self* subscale and from 0.797 to 0.803 in the case of the *compassion action orientation* subscale).

### Reliability analysis

Both Cronbach’s alpha and McDonald’s omega were calculated for each subscale. Cronbach’s alpha assumes unidimensionality but also assumes that the measurement model is essentially tau-equivalent (Graham, 2006). McDonald’s omega also assumes unidimensionality but, less stringently, assumes a congeneric measurement model (see Table 1).

**TABLE 1:** Subscale reliability coefficients.

Subscale	Cronbach’s alpha	McDonald’s omega
Investing the self	0.669	0.666
Mindfulness	0.738	0.735
Recognition of emotion	0.831	0.828
Gaining and communicating an empathic understanding	0.868	0.871
Caring with kindness	0.829	0.831
Compassion action orientation	0.803	0.802

Source: Visser, M. (2020). *The development and psychometric evaluation of a medical practitioner compassion competency questionnaire*. Doctoral dissertation, Stellenbosch University. Retrieved from http://hdl.handle.net/10019.1/108023

The reliability of the *investing the self* subscale and, although to a somewhat lesser extent, the *mindfulness* subscale gave reason for concern. The dimension scores on *the investing the self* subscale should therefore be interpreted with great caution. Exactly what caused the poor internal consistency is difficult to definitively explain, given the fact that the unidimensionality assumption has been corroborated for this subscale.

### Test of multivariate normality

The medical practitioner compassion competency construct was conceptualised in terms of six correlated latent competency dimensions. Specific items were written to reflect each of the latent compassion competency dimensions. To determine whether this design intention succeeded, a first-order measurement model was fitted.

The MPCCQ item dataset was first tested for multivariate normality via PRELIS, testing the statistical significance of Mardia’s measure of multivariate skewness and kurtosis (Mardia, 1970). The null hypothesis that the item distribution follows a multivariate normal distribution had to be rejected (*p* < 0.05). An attempt was made to normalise the data which failed. The skewness and kurtosis Chi-square value increased from 716.841 to 948.015, intensifying the initial deviation from normality.

**FIGURE 3:**
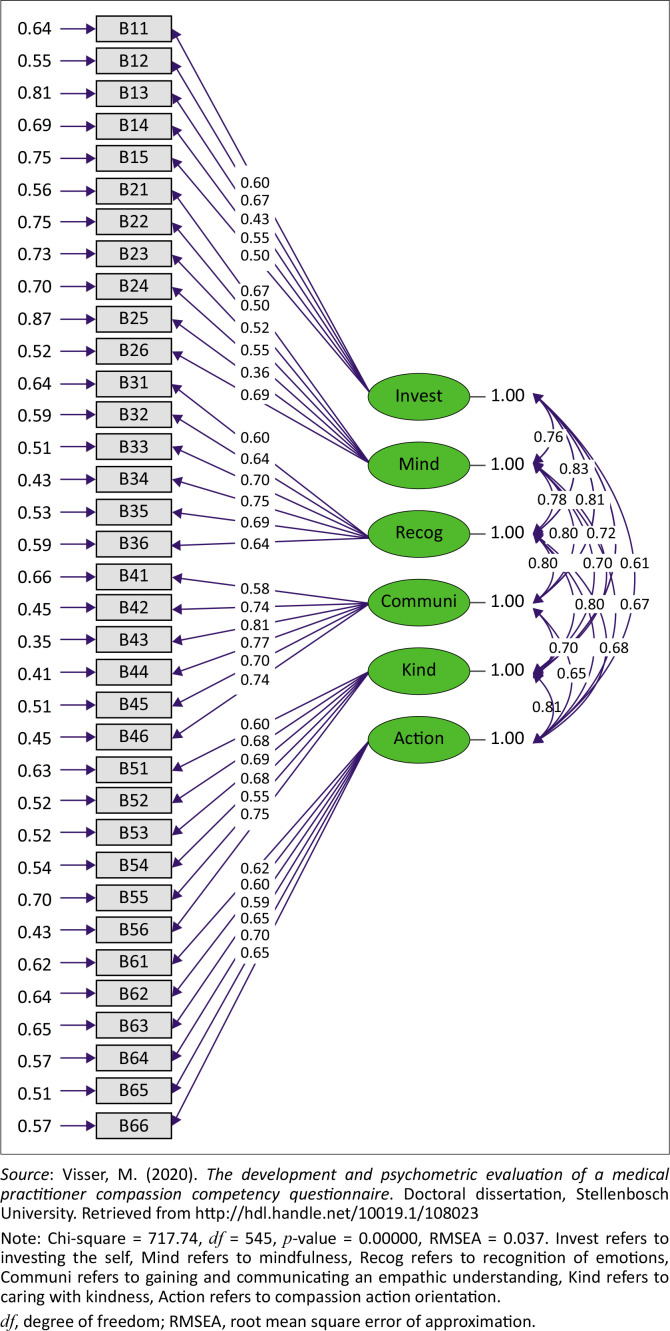
Medical practitioner compassion competency measurement model (completely standardised solution).

Thus, the MPCCQ measurement model was fitted to the original non-normalised item dataset, using robust maximum likelihood (RML) estimation. The measurement model converged after 13 iterations with an admissible solution. The completely standardised fitted medical practitioner compassion competency model is shown in Figure 3.

### Goodness of fit statistics

Model fit indices are reported in Table 2. The Satorra-Bentler Chi-square (χ^2^) calculated due to the use of the RML estimation procedure, and shown in Table 2, returned a significant statistical value of 717.7365 (*p* ˂ 0.05). The exact fit null hypothesis (*H*_0_: RMSEA = 0) was consequently rejected. The sample estimate for the root mean square error of approximation indicated good model fit (RMSEA = 0.037, *p* > 0.05). The probability of obtaining a sample RMSEA estimate of 0.037 under the close fit null hypothesis (*H*_0_: RMSEA = 0.05) was sufficiently large (*p* > 0.05) not to reject the close fit null hypothesis (Visser, 2020).

**TABLE 2:** Goodness of fit statistics for the medical practitioner compassion competency measurement model.

Fit statistic	Value
*df*	545
**Minimum fit function**	
Chi-square	840.8589
*p*	0.00
**Normal theory weighted least squares**	
Chi-square	846.1498
*p*	0.00
**Satorra-Bentler scaled**	
Chi-square	717.7365
*p*	0.0000
Estimated non-centrality parameter (NCP)	172.7365
90% CI for NCP	(107.3456; 246.2283)
Minimum fit function value	3.5934
Population discrepancy function value (F_0_)	0.7382
90% CI for F_0_	(0.4587; 1.0523)
Root mean square error of approximation (RMSEA)	0.03680
90% CI for RMSEA	(0.02901; 0.04394)
*P*-value for test of close fit (RMSEA < 0.05)	0.9992
Expected cross-validation index (ECVI)	3.7937
90% CI for ECVI	(3.5143; 4.1078)
ECVI for saturated model	5.3846
ECVI for independence model	66.2580
Chi-square for independence model with 595 *df*	15434.3680
Independence AIC	15504.3680
Model AIC	887.7365
Saturated AIC	1260.0000
Independence CAIC	15660.4535
Model CAIC	1266.8013
Saturated CAIC	4069.5389
Normed fit index (NFI)	0.9535
Non-normed fit index (NNFI)	0.9873
Parsimony normed fit index (PNFI)	0.8734
Comparative fit index (CFI)	0.9884
Incremental fit index (IFI)	0.9884
Relative fit index (RFI)	0.9492
Critical *N* (CN)	204.6802
Root mean square residual (RMR)	0.05494
Standardised RMR	0.05441
Goodness of fit index (GFI)	0.8288
Adjusted goodness of fit index (AGFI)	0.8020
Parsimony goodness of fit index (PGFI)	0.7169

Source: Visser, M. (2020). *The development and psychometric evaluation of a medical practitioner compassion competency questionnaire*. Doctoral dissertation, Stellenbosch University. Retrieved from http://hdl.handle.net/10019.1/108023

CI, confidence interval; *df*, degree of freedom; AIC, Akaike information criterion; CAIC, consistent Akaike information criterion.

The expected cross-validation index (ECVI) indicates the difference between the reproduced sample covariance matrix resulting from fitting the model on the medical practitioner sample and the expected covariance matrix that could be achieved in an independent sample of the same sample size of medical practitioners. Because the ECVI for the fitted model is smaller (3.7937) than the ECVI for the independent model (66.2580) and also smaller than the ECVI value of the saturated model (5.3846), the fitted model is expected to fit better in an independent sample (Visser, 2020).

The independence AIC (15504.3680), model AIC (887.7365), saturated AIC (1260.0000), independence consistent Akaike information criterion (CAIC) (15660.4535), model CAIC (1266.8013) and lastly saturated CAIC (4069.5389) all returned the smallest value for the fitted model thus indicating a good-fitting model. In addition, the normed fit index (NFI) (0.9535) and the CFI (0.9884) both indicated good model fit (Visser, 2020).

The root mean square residual (0.05494) and the SRMR (0.05441) also indicated a good-fitting model. The MPCCQ measurement model demonstrated good fit, as indicated by the basket of fit indices in Table 2 (Visser, 2020).

### Completely standardised lambda-X matrix

To determine the validity of the test items, as indicators of the level of competence shown by the medical practitioners on the specific latent compassion competencies the items were designated to reflect, the completely standardised solution for the factor loading matrix (lambda-X matrix) was interpreted. The completely standardised factor loadings for all items were statistically significant (*p* < 0.05) and all exceeded the cut-off value of 0.50, except for item B13 (λ_31_ = 0.4349) and item B25 (λ_10,2_ = 0.3611). These two items had completely standardised factor loadings which were below 0.50, but still above 0.30, which was still regarded as acceptable: not satisfactory but not totally unacceptable. The two items with somewhat lower than desired completely standardised factor loadings (B13 and item B25) were therefore somewhat less sensitive to changes in the latent compassion competency they were designated to reflect compared to other items in the lambda matrix (Visser, 2020).

### Completely standardised theta-delta matrix

The completely standardised measurement error variance-covariance (theta-delta) matrix indicated that all measurement error variances were statistically significant (*p* < 0.05), but two exceeded the critical value of 0.75 implied by the critical completely standardised factor loading of 0.50 that was decided upon. Item B13 and item B25 were the only two problematic items in the sense that more than 75% of the variance in the test item was the result of systematic and random measurement error; thus variance that is not explained by the latent dimension they were designated to reflect (Visser, 2020).

### Squared multiple correlations

Squared multiple correlations (*R*^2^) of the item indicators were examined. Almost all (*n* = 33 out of 35) item indicators provided valid explanations of the latent dimensions they were designed to reflect (*R*^2^ ≥ 0.25) when using the critical cut-off value set for the completely standardised factor loadings as a basis. Only item B13 (‘Medical practitioner personal disclosure/exposure’) and item B25 (‘Registering the current moment accurately’) had squared multiple correlations which were below the cut-off value (*R*^2^ < 0.25) (Visser, 2020).

### Standardised phi matrix

Correlations in the standardised phi matrix between all latent dimensions were statistically significant (*p* < 0.05). The completely standardised phi matrix indicated no excessively high correlations. A correlation was deemed excessively high in the current study if the value exceeded a cut-off of 0.90 (Visser, 2020).

### Test discriminant validity

None of the latent compassion dimensions measured by the MPCCQ correlated excessively highly (ɸ_kp_ ≥ 0.90) in the sample. The possibility, however, still existed that the parametric value of one or more ɸ_kp_ could be equal to 1 while the corresponding sample estimates were smaller because of sampling error. Fifteen 95% confidence intervals were consequently calculated (Visser, 2020). The confidence intervals indicate the upper and lower bound ɸ_kp_ values between which the parametric ɸ_kp_ can be expected with 95% confidence. The key question is whether any interval contains the value of 1. If not, it can be argued with 95% confidence that none of the ɸ_kp_ correlations in general were equal to 1, which then implies discriminant validity. None of the 15 confidence intervals contained a parametric ɸ_kp_ estimate that approximated 1. The MPCCQ thus successfully measured the six latent compassion dimensions as qualitatively distinct, but correlated, latent competencies (Visser, 2020).

In addition to the confidence intervals, a more stringent criterion was used to evaluate discriminant validity: the average variance extracted (AVE) measure (Diamantopoulos & Siguaw, 2000). Farrell (2010) is of the opinion that the MPCCQ dimensions should account for more variance in the subscale items than measurement error (Visser, 2020). These dimensions should also account for more variance in the subscale items that represent them than they account for in each other. None of the 15 squared inter-latent variable correlations were smaller than both the AVE values associated with the latent variable pairs being correlated, as shown in Table 3.

**TABLE 3:** Average variance extracted calculated for each latent compassion dimension and squared inter-latent variable correlations.

Competency dimensions	Invest	Mind	Recog	Communi	Kind	Action	AVE
Invest	1.0000000000	-	-	-	-	-	0.310530054
Mind	0.6105859600‡	1.000000000	-	-	-	-	0.314268359
Recog	0.6844252900‡	0.580644000‡	1.0000000	-	-	-	0.450859760
Communi	0.6588568900‡	0.668796840‡	0.7471874‡	1.00000000	-	-	0.530005293
Kind	0.5181120400‡	0.553684810‡	0.6404801‡	0.52969284§	1.000000000	-	0.443352443
Action	0.3724660900§	0.449168040‡	0.4598196‡	0.42120100§	0.655776040‡	1.000000	0.406998023

**AVE**	**0.3105300540**	**0.314268359**	**0.4508598**	**0.53000529**	**0.443352440**	**0.406998**	-

Note: INVEST refers to investing the self, MIND refers to mindfulness, RECOG refers to recognition of emotions, COMMUNI refers to gaining and communicating an empathic understanding, KIND refers to caring with kindness, ACTION refers to compassion action orientation.

‡ , PHI^2^ > both AVE;

§ , PHI^2^ > one AVE.

The finding that the AVE was less than the squared inter-latent variable correlation implied that the unique part of the latent variable had not been adequately measured. Three of the squared inter-latent variable correlations were smaller than the AVE value associated with one of the latent variables in the pair of variables being correlated (shown * in Table 3) (Visser, 2020). One of the latent variables involved in three pairs of inter-latent variable correlations therefore accounted for less variance in each other than the average variance it accounted for in the items designated to reflect it. Twelve of the 15 squared inter-latent variable correlations were larger than both the AVE values associated with the latent variable pairs being correlated (shown ** in Table 3) (Visser, 2020).

Farrell’s (2010) proposed criterion, however, should be critically examined. What is of note is that the subscales are evaluated on the average variance that the designated latent dimension explains in its items, rather than on the validity and reliability of the subscale score. Systematic and random measurement error tends to generally afflict individual subscale items. The designated latent dimension is generally expected to explain relatively little variance in each individual item. The expectation of this research study was that the latent dimension should explain at least 25% of the variance in the individual items earmarked to reflect it. The postulation is that the designated latent dimension is the only common source of systematic variance across items. When responses to these error-prone items are combined into a linear composite, the reliability and validity of the dimension score are substantially higher than the reliability and validity of the individual items (Ghiselli et al., 1981; Nunnally, 1978; Visser, 2020).

### Comprehensive MPCC LISREL model

The fact that the MPCCQ measurement model showed close fit and the fact that the parameter estimates were largely favourable constitute evidence that the intention to measure six latent variables via 37 items largely succeeded. It does not, however, provide strong evidence that the six latent dimensions reflected by the items are the latent compassion competencies in terms of which the compassion competency construct was conceptualised. The connotative meaning of a construct, in part, lies in its internal structure. Figure 1 depicts the internal structure attributed to the medical practitioner’s compassion competency construct. If the MPCCQ provides construct-valid measures of the medical practitioner compassion competency construct, the structural model depicted in Figure 1 should fit that MPCCQ item data and the hypothesised paths should be statistically significant (*p* < 0.05),^3^ The comprehensive LISREL model achieved close fit (*p* > 0.05). The close fit of the measurement model, combined with the close fit of the comprehensive LISREL model, allowed the inference of acceptable fit for the structural model. Moreover, only one of the hypothesised paths between the latent compassion dimensions was not statistically significant (*p* > 0.05), namely the path from *investing the self* to *gaining and communicating an empathic understanding*. This means that *investing the self* does not have a direct effect on *gaining and communicating an empathic understanding*. This implies that a medical practitioner cannot simply invest themself in the medical encounter, hoping that this in and by itself will result in successfully gaining and communicating an empathic understanding (Visser, 2020). Rather the effect of authentically *investing the self* in the practitioner-patient encounter on *gaining and communicating an empathic understanding* is indirect. The effect of investing the self in the practitioner-patient encounter on *gaining and communicating an empathic understanding* is mediated by *mindfulness* and *recognition* of emotions. A medical practitioner investing themself in the patient-practitioner relationship will, because of this, tend to be more psychologically and physically present in the moment (*mindfulness*) and more competent at recognising emotions. The practitioner will, because of both the increased competence at being mindful as well as recognising emotions, be more competent at *gaining and communicating an empathic understanding* (Visser, 2020). The close fit combined with the statistical significance (*p* < 0.05) of seven of the eight conceptualised paths provided further support for the construct validity of the MPCCQ.

## Discussion

Collectively, the findings on the MPCCQ measurement and structural models served to support the hypothesis that construct-referenced inferences about medical practitioners’ standing on the latent dimensions of the medical practitioner compassion competency construct, as derived from the dimension scores, are permissible. The reliability findings on the *investing the self* subscale, however, indicate the need for caution when interpreting this dimension score.

In recent years, the measuring of compassion within healthcare has received increased scientific interest. Measuring compassion is a challenge; but given the need for compassionate patient-centred healthcare, a clinically relevant and psychometrically robust instrument for measuring compassion in healthcare and educational settings is no longer a choice, but a necessity.

This study contributes to the public healthcare sector by providing a psychometrically sound instrument for measuring medical practitioner compassion as a multidimensional competency.^4^ The current self-assessment version of the MPCCQ can be adapted into an other-rater version, allowing patients and other professionals to evaluate medical practitioner compassion. This research offers a range of possibilities not only for medical practitioners but also for professional healthcare institutions, accredited bodies and educational institutions. It allows for synergy across interdisciplinary fields such as psychology and human resource management in healthcare by contributing to areas such as performance management, recruitment and selection as well as learning and development for medical practitioners (Visser, 2020).

### Limitations

The position that the MPCCQ provides construct-valid assessments of the medical practitioner compassion competency as constitutively defined has thus far survived the opportunities to refute this position. The cautious utilisation of the MPCCQ thus seems warranted. However, unanswered questions still exist.

The connotative meaning of a construct also lies in the manner in which the construct is embedded in a larger nomological net of latent variables. This aspect of the connotative meaning of the medical practitioner compassion competency construct still needs to be explicated. Once the constitutive definition of the compassion construct has been extended in this manner, the construct validity of the MPCCQ can be further evaluated by evaluating the fit of the structural model reflecting the manner in which the compassion construct is understood to be embedded in a larger nomological net and testing the statistical significance of the structural parameter estimates.

The six latent compassion competency dimensions correlate to some degree in the measurement model. They, therefore, to some degree, share variance. The inter-latent compassion competency dimension correlation matrix can be explained by a second-order factor structure of one or more second-order factors. The second-order factors represent the themes shared by the first-order factors. The second-order factor structure of the MPCCQ has not as yet been identified.^5^ Once it has been identified, two important interrelated questions will be raised. The first is to what extent the explained item variance can be attributed to the themes unique to the first-order factors, and to what extent the second-order factors representing the item responses are determined by the themes shared by the first-order factors (i.e. the second-order factors). This question can be answered by fitting a second-order measurement model in which the first-order compassion factors load on the identified second-order factors and transforming the completely standardised solution to a bifactor model via a Schmid-Leiman transformation (Brown, 2006). The ideal would be that the residualised first-order factors do not account for very small proportions of the explained item variance. If the themes unique to the first-order factors account for little of the explained item variance, the interpretation of the first-order dimension scores becomes problematic.

The second question flows from the realisation that subscale items, to some degree, reflect variance in a theme unique to that subscale (the residualised first-order factor) and to some degree reflect variance in a theme shared with the items of one or more other subscales (the second-order factor on which the first-order factors load). The dimension score calculated from the subscale items is therefore always, to borrow an oenological term, a blend of the theme unique to the first-order factor (i.e., the residualised first-order factor) and the theme shared by the first-order factors loading on the same second-order factor. The question is whether, in practical assessment situations, it would be possible to obtain ‘single-cultivar’ measures of the residualised first-order factors and the second-order factors.

Scores obtained on a psychological test or questionnaire cannot be meaningfully interpreted in the absence of norms. Construct-referenced norms have, thus far, not been developed for the MPCCQ. This seriously restricts the practical use of the MPCCQ.^6^

A further question that thus far has not been answered is whether the MPCCQ measures the same construct in different gender and racial groups, and if so, whether it measures the construct in the same manner. The latter part of the question essentially asks whether gender or race explains variance in the MPCCQ item responses either as a main effect or in interaction with the compassion competency dimension that is not explained by the compassion competency dimension. If group membership does explain variance in the item responses that is not explained by the compassion competency dimension, the same item score cannot be interpreted in the same way across gender and/or racial groups. Both construct and item bias can be evaluated via multigroup confirmatory factor analysis (CFA), in which, in addition to the slope, the intercept of the regression of the item on the latent variable is also modelled.

### Recommendations for future research

Recommendations for further research include the revision of the *investing the self* subscale and the possible writing of additional items to improve the reliability of the subscale. In addition, further validation studies of the MPCCQ on new samples are recommended.

Medical practitioner compassion also needs to be understood in the way it is embedded in a larger nomological network of competency potential, competencies and competency outcome latent variables. This structural model reflecting the manner in which this construct is believed to be embedded in a larger nomological network needs to be developed. The fit of the model and the evaluation of the statistical significance of the structural model parameter estimates need to be determined.

Concerning item analysis, the recommendation is that item response theory should be used in addition to the classical measurement theory item analysis that was used for this study. This will assist in locating the items on the latent trait scale and their ability to discriminate at the important points on the latent trait scale. In addition, the derivation of a second-order factor structure for the MPCCQ via an unrestricted exploratory factor analysis of the phi matrix should be explored. Also, the evaluation of the fit of a second-order MPCCQ measurement model reflecting the derived second-order factor structure and the mathematical transformation of the completely standardised solution to a bifactor measurement model via a Schmid-Leiman transformation should be considered.

The MPCCQ is a self-rater instrument that assists medical practitioners in understanding their own compassion profile. The next step would be to adopt this questionnaire into an other-rater version, allowing patients and other professionals to evaluate medical practitioner compassion, as well as the validation of the other-rater MPCCQ. Construct-referenced norm tables for both these two instruments, however, still need to be developed. A final recommendation would be the investigation of measurement bias across gender and racial groups via multigroup CFA.

In conclusion, this research addressed an unmet need for a psychometrically validated instrument that comprehensively measures the construct of medical practitioner compassion competency in the healthcare setting.

## Footnotes

1 It is acknowledged that the connotative meaning of a construct also lies in the manner in which the construct is embedded in a larger nomological network of latent variables. The connotative meaning of the medical practitioner compassion competency construct therefore needs to be further explicated via research that embeds the construct in a larger nomological net of antecedent and outcome latent variables.

2 This position could possibly raise a few eyebrows. Kellaghan (2010) defines evaluation research ‘as a form of disciplined and systematic inquiry that is carried out to arrive at an assessment or appraisal of an object, program, practice, activity, or system with the purpose of providing information that will be of use in decision making’.

3 The connotative meaning of the medical practitioner compassion competency construct also lies in the manner in which the construct is structurally embedded in a larger nomological network. Further evidence in support of the construct validity of the MPCCQ will be gained if a structural model reflecting this stance fits and the path coefficients are statistically significant (*p* < 0.05).

4 The MPCCQ is available Visser (2020). The fact that construct-referenced norms still need to be developed currently limits the practical use of the instrument.

5 Explication of the second-order factor structure and the manner in which the first-order MPCCQ factors load onto the second-order factor/factors will further deepen the connotative meaning of the medical practitioner compassion competency construct.

6 Neither has criterion-referenced norms been developed for the MPCCQ.
